# Asoprisnil, a Selective Progesterone Receptor Modulator (SPRM), Inhibits Melanosome Export in B16F10 Cells and HEMn-DP Melanocytes

**DOI:** 10.3390/molecules25163581

**Published:** 2020-08-06

**Authors:** Shilpi Goenka, Sanford R. Simon

**Affiliations:** 1 Department of Biomedical Engineering, Stony Brook University, Stony Brook, NY 11794-5281, USA; sanford.simon@stonybrook.edu; 2 Department of Pathology, Stony Brook University, Stony Brook, NY 11794-5281, USA; 3 Department of Biochemistry and Cellular Biology, Stony Brook University, Stony Brook, NY 11794-5281, USA

**Keywords:** asoprisnil, selective progesterone receptor modulator, drug-repurposing, B16F10 cells, HEMn-DP cells, anti-melanogenic, melanosome export

## Abstract

Previous studies have reported that estrogen hormone promotes melanogenesis while progesterone inhibits it. A selective estrogen receptor modulator (SERM), tamoxifen, has been shown to promote melanogenesis; however, to date, there have been no reports on the effects of a selective progesterone receptor modulator (SPRM) on melanogenesis. In the present study, we hypothesized that asoprisnil (AP), a SPRM, inhibits melanogenesis. AP was tested for cytotoxicity to B16F10 mouse melanoma cells for screening the nontoxic concentrations using MTS cytotoxicity assay. Extracellular and intracellular melanin levels were estimated at nontoxic concentrations of AP. To evaluate the direct effect of AP on tyrosinase enzyme, tyrosinase activity and copper chelating activities were measured. Next, the effects of AP on melanogenesis were tested in normal human melanocytes, neonatal, darkly pigmented (HEMn-DP). Our results demonstrate that AP was nontoxic at a concentration range of 10–50 μM in B16F10 cells; AP at 50 μM significantly suppressed extracellular melanin levels comparable to kojic acid at 500 μM, with no significant effect on intracellular melanin levels. The mechanism of melanogenesis inhibition was studied to assess if AP downregulated tyrosinase activity in cell lysates or in a cell-free system. However, AP was found to increase intracellular tyrosinase activity without any effect on tyrosinase enzyme activity or copper chelating activity in a cell-free system, indicating that AP inhibits melanogenesis by mechanisms other than direct effects on tyrosinase enzyme activity. The capacity of AP to inhibit melanosome export was further validated in HEMn-DP cells; AP significantly suppressed dendricity at concentrations of 20 and 30 μM in the absence of effects on melanin synthesis or intracellular tyrosinase activity. In addition, AP was nontoxic to human keratinocytes (HaCaT) at these concentrations, validating its safety for topical use. Taken together, our preliminary results demonstrate that AP might be repurposed as a candidate therapeutic for treatment of hyperpigmentation disorders via a unique mechanism, which encompasses a selective inhibition of melanosome export.

## 1. Introduction

Melanin pigment is synthesized in melanocytes located in the basal layers of the epidermis and is exported via dendrites to keratinocytes where it accumulates perinuclearly and causes skin coloration. Typically, a single melanocyte transfers melanin pigment to up to 36 keratinocytes via its arborized dendrites [1]. Melanin provides protection against harmful UV radiation and DNA damage and scavenges reactive oxygen species [2]. Hyperpigmentation is caused by over-accumulation of melanin pigment in the skin and is associated with skin conditions such as melasma, solar lentigo (age spots), ephelis, and post-inflammatory hyperpigmentation (PIH), which can compromise aesthetic appearance [3]. Tyrosinase is the key rate-limiting enzyme in melanin production in cells; it catalyzes the hydroxylation of tyrosine to L-DOPA (3,4-dihydroxyphenylalanine) and subsequent oxidation to dopaquinone [4]. The most popular skin-depigmenting compounds are tyrosinase inhibitors such as hydroquinone and kojic acid (KA), which exhibit serious side effects; KA causes contact allergy [5] and tumorigenesis [6], while hydroquinone exhibits carcinogenicity [7]. Due to these limitations, there is an active interest in the search for novel compounds for the reduction in skin pigmentation that do not exhibit adverse effects and that have a relatively safe profile. As several of the skin-whitening compounds are tyrosinase inhibitors, alternative targets to inhibit melanogenesis have been actively researched. Some of these are directed to later steps in the melanogenesis cascade, such as the export of melanin pigment via dendrites, as after synthesis in the cells, melanin is secreted by melanocytes and taken up by keratinocytes via various pathways [8,9]. Dendrites are actin and microtubule-containing structural features of melanocytes and are key conduits of the export of melanin pigment to keratinocytes [10]; compounds that can promote or inhibit dendricity can be used to control pigmentation disorders.

The role of hormones in regulating melanogenesis has been previously documented [11]. For example, progesterone inhibited, while estrogen stimulated, melanogenesis in human melanocytes [12,13]. Selective progesterone receptor modulators (SPRMs) belong to the novel class of synthetic steroids, which exhibit antagonist, agonist, or mixed effects upon progesterone receptor (PR) binding in progesterone target tissues in vivo [14]. Asoprisnil (AP) is a 11β-benzaldoxime-substituted steroidal SPRM, which has been used in clinical studies for treatment of uterine leiomyomata and endometrial neoplasms [15,16,17,18]. Furthermore, the antiproliferative effects of AP were selective to uterine leiomyoma cells, and normal myometrial cells were not affected [19]. Besides AP, other SPRMs such as telapristone and ulipristal acetate have been used for management of uterine fibroids and developed for clinical use with varying degrees of success [20]. For example, while AP was historically one of the earliest SPRMs to be studied for two randomized trials, its use was halted in 2007 due to changes in priorities of the corporate sponsor. Telapristone was developed concurrently but, due to liver toxicity, its use was abandoned in 2009 but restarted at lower doses. Ulipristal acetate was the only SPRM to be approved by the US Food and Drug Administration (FDA) in 2010 [21]. However, AP differs from ulipristal acetate and other SPRMs as it has shown to demonstrate a higher level of progesterone agonist vs. antagonist activity in animal models [22] and, hence, was selected in the current study out of other SPRMs. In addition, the most recent study in 2019 highlighted that AP does not induce liver toxicity and has an appreciable safety profile [23].

In our previous study, we reported anti-melanogenic effects of the anti-rheumatoid and anticancer drug auranofin, which inhibited melanin biosynthesis in both B16F10 and MNT-1 cells [24]. Previous studies have reported melanogenesis inhibition by anticancer drugs, for example, an anticancer drug imatinib mesylate [25], and an FDA-approved synthetic testosterone drug danazol [26] inhibited melanogenesis by suppressing melanin biosynthesis in B16F10 cells. Another study reported that tamoxifen, a selective estrogen receptor modulator (SERM) used for treatment of breast cancer, increased melanogenesis by augmenting melanin secretion in human melanocytes from darkly pigmented neonatal donor (HEMn-DP) cells [27]. However, to date, no study exists in which a SPRM has been studied for its effects on melanogenesis. As nature-based compounds need to undergo clinical trials for testing their safety and toxicity in vivo, there is an unmet need for nontoxic skin depigmenting agents with a well-established safety profile, which could help escalate their use for clinical dermatological disorders such as hyperpigmentation and melasma. In this report, we explored AP for repurposing for the treatment of hyperpigmentation disorders and tested AP to evaluate its depigmenting capacity in B16F10 mouse melanoma cells, and we also validated our results in primary human melanocytes.

## 2. Results

### 2.1. AP Inhibited Extracellular Melanogenesis at Nontoxic Doses in B16F10 Cells

**Figure 1:**
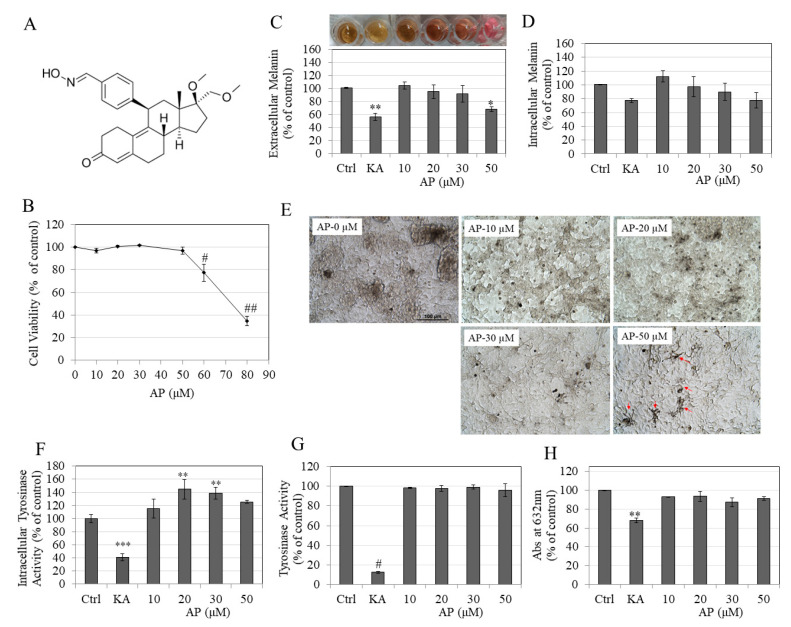
(**A**) Chemical structure of asoprisnil (AP); (**B**) Viability of B16F10 mouse melanoma cells treated for 72 h in the presence of different concentrations of AP as measured by the reduction in the tetrazolium salt MTS. Control wells were treated with 0.4% DMSO; # *p* = 0.0027; ## *p* < 0.0001; (**C**) Extracellular melanin in cultures of B16F10 cells treated with AP for 72 h; panel shows pictures of the corresponding cell culture medium; * *p* < 0.05; ** *p* < 0.01 vs. Control; one-way ANOVA with Dunnett’s test; (**D**) intracellular melanin levels; kojic acid (KA) at 0.5 mM was used as a positive control; (**E**) photomicrographs of B16F10 cells treated with different concentrations of AP; objective magnification is 20x; melanin aggregation is visible in AP-50 µM group (red arrows); (**F**) effect of AP on intracellular tyrosinase activity in B16F10 cells; (**G**) direct effect of AP on tyrosinase activity using mushroom tyrosinase enzyme; (**H**) effect of AP on copper chelation activity measured by absorbance at 632 nm; KA was used as a positive control at 500 µM in all assays; ** *p* < 0.01; *** *p* < 0.001, and # *p* < 0.0001 vs. Ctrl by one-way ANOVA followed by Dunnett’s post-hoc test. All data are mean ± SEM of at least three independent experiments, except for (**F**), which is mean ± SEM pooled from two separate experiments, and (**H**), which is mean ± SEM of at least two independent experiments.

AP (chemical structure, Figure 1A) was found to be nontoxic up to concentrations of 50 µM to B16F10 cells, while the higher concentrations of 60 and 80 µM elicited significant cytotoxicity with a reduction in viability by 22.54% and 65.20%, respectively (Figure 1B). AP at a concentration range of 10–50 µM was thus further assayed to test its effects on melanogenesis. Our results showed that AP reduced melanin secretion in the culture medium in a concentration-dependent manner; a significant reduction of 32% was observed at AP-50 µM with a visible reduction in color of the culture supernatant as compared to untreated culture (Figure 1C; panel). The results on intracellular melanin showed that treatment with AP showed a trend for the reduction in the cellular melanin levels for AP at 50 µM, but no significance was reached (Figure 1D).

Additionally, the photomicrographs of cells treated with AP showed a tendency of accumulation of melanin pigment in clusters, especially at AP-50 µM (Figure 1E), which showed that AP may inhibit melanin release in the medium by inducing aggregation of melanosomes. Taken together, these results demonstrate that AP selectively inhibits extracellular melanogenesis by targeting melanosome export.

### 2.2. AP Enhanced Intracellular Tyrosinase Activity Without Any Effect on Direct Tyrosinase Activity

AP showed a biphasic response by increasing intracellular tyrosinase activity at all concentrations, which significantly peaked by 44.67% and 38.87% at concentrations of 20 and 30 µM, respectively, as compared to control (Figure 1F). AP at a concentration of 50 µM showed an increase of 25.55% but was not significant from the control group. By contrast, the results of mushroom tyrosinase activity showed no change at any dose of AP treatment (Figure 1G). Overall, these results highlight that AP inhibits extracellular melanogenesis by pathways not involving any action on either the direct or the intracellular tyrosinase activity.

### 2.3. AP Does not Show Copper Chelation

We next tested if AP may show any copper chelation capacity directly in the absence of cells, as several anti-melanogenic agents exhibit copper chelation activity. However, AP did not show any copper chelation activity at any concentrations (Figure 1H). KA, used as a positive control, significantly chelated by 32.21%. Overall, this result shows that AP does not inhibit melanogenesis by chelating the copper in tyrosinase enzyme in vitro.

### 2.4. AP Did Not Affect Intracellular Melanin at Nontoxic Concentrations in Human Melanocytes

**Figure 2:**
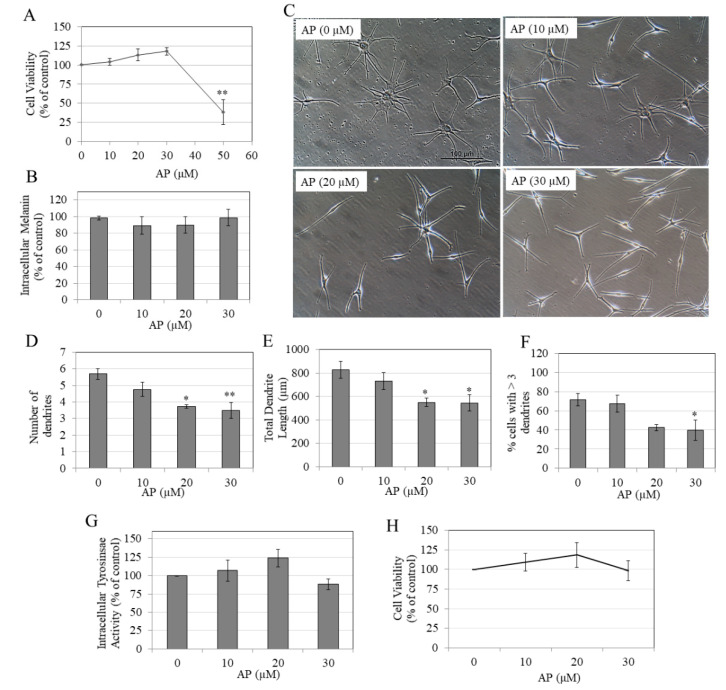
(**A**) Viability of HEM-DP cells treated with AP at various doses for 5 d; ** *p* < 0.01 vs. Control group; one-way ANOVA with Dunnett’s test; (**B**) intracellular melanin levels; and (**C**) representative phase-contrast images of cells treated with AP (0–30 µM) taken at 20x objective magnification; melanocyte dendricity quantification by parameters: (**D**) Number of dendrites, (**E**) total dendrite length, and (**F**) % cells with >3 dendrites; a total of ~100 cells were counted for each treatment group; * *p* < 0.05 and ** *p* < 0.01 by one-way ANOVA with Dunnett’s test; (**G**) Intracellular tyrosinase activity in cultures of HEMn-DP cells treated with AP for 5 d; (**H**) viability of human keratinocytes (HaCaT) treated with AP at various doses for 5 d. All data are mean ± SEM of at least three independent experiments.

In order to validate the anti-melanogenic activity of AP in primary human melanocytes, we first tested AP for cytotoxicity in HEMn-DP cells over a duration of 5 d to rule out any contribution of cytotoxicity on melanogenesis inhibition. Our results showed that AP was significantly toxic at 50 µM (reduction in viability by 61.72%, Figure 2A), while lower concentrations were nontoxic; hence, these concentrations were used for subsequent analysis on melanogenesis. Our results on melanogenesis showed that AP did not inhibit melanin synthesis at any concentration in HEMn-DP cells (Figure 2B).

### 2.5. AP Inhibits Dendricity in Human Melanocytes

We next tested if AP may exhibit any inhibitory effect on one or more subsequent steps in melanogenesis, including the export of melanosomes via melanocyte dendrites, as our earlier results showed that AP did not affect melanin synthesis in these cells. The control group contained cells with multiple dendrites (Figure 2C), which were not reduced in number in AP-treated groups at the lowest concentration of 10 µM; however, a significant suppression in dendrite number was achieved at higher concentrations with a reduction of 34.89% and 38.72% in cells treated with AP at 20 and 30 µM, respectively (Figure 2D). Moreover, our results on total dendrite length showed that AP significantly suppressed the total dendrite length by 33.60% and 34.06% at concentrations of 20 and 30 µM, respectively, as compared to the control group (Figure 2E). Additionally, the % cells with >3 dendrites were reduced by 28.90% and 31.58% at concentrations of 20 and 30 µM, respectively, with significance reached for 30 µM (Figure 2F).

Altogether, these results showed that in human melanocytes, AP demonstrates a robust capacity to downregulate melanosome export by inhibiting dendrite number and length, causing a reduction in the % of multipolar dendrites; this would lead to a reduction in the export of melanin to keratinocytes, thereby inhibiting pigmentation.

### 2.6. AP Does Not Inhibit Intracellular Tyrosinase Activity in Human Melanocytes

AP did not inhibit intracellular tyrosinase activity at any concentration; a trend for increase was noted, which was not significant (Figure 2G).

### 2.7. AP is Nontoxic to Human Keratinocytes

We next conducted cytotoxicity assay in human keratinocyte (HaCaT) cells over a concentration range (0–30 µM) to determine if AP might be nontoxic to HaCaT cells at this concentration range where it inhibited melanogenesis in HEMn-DP cells, as obtained in our earlier results; this would confirm that AP was effective as well as nontoxic for topical use. Our results showed that AP was nontoxic in the concentration range of 10–30 µM in the duration of 5 d (Figure 2H), validating that AP remained nontoxic to keratinocytes at the concentration and duration range where it inhibited melanocyte dendricity in our earlier results.

## 3. Discussion

Our results demonstrate that the SPRM AP demonstrates the capacity to inhibit melanogenesis by selectively inhibiting one or more subsequent steps of melanosome export in the absence of the effect on melanin biosynthesis in both B16F10 mouse melanoma cells and primary human melanocytes. Furthermore, our results also demonstrate that AP is nontoxic to keratinocytes at the low micromolar doses at which it inhibited melanogenesis. Several earlier reports evidence the role of hormones in affecting skin pigmentation [11,12,28], and it has been documented that progesterone might be useful in inclusion in oral contraceptives to counteract the development of melasma that is often attributable to estrogens [29]. A higher expression of progesterone receptor (PR) has been documented in epidermal skin lesions in contrast to healthy skin [30]. A previous study has documented the presence of classical progesterone receptors in primary human melanocytes by RT-PCR (Reverse Transcription-Polymerase Chain Reaction) and immunocytochemical methods [31]. The biological action of progesterone can be mediated either by binding to nuclear receptors (classical pathway) or by binding to a membrane receptor and activation of an intracellular signaling cascade and transcription (non-classical pathway). The possibility that SPRMs such as AP might function by the non-classical pathway not involving PR binding cannot be ruled out, and future studies to assay for changes in the transcription level of genes involved in melanogenesis are warranted. In a previous report, which was conducted on tamoxifen, a SERM, the authors noted an enhancement in melanin secretion in human melanocytes, which led to increased melanogenesis [27]. Interestingly, in our study, we tested AP, which is a SPRM and significantly suppressed melanin secretion without affecting melanin synthesis in B16F10 mouse melanoma cells; this result was validated in primary human melanocytes where AP inhibited melanosome export, which was quantitated by dendricity without any effect on melanin biosynthesis. Our findings of melanosome aggregation by AP in B16F10 cells are similar to results of another study where a natural compound hesperidin induced melanosome aggregation accompanied by a reduction in melanin secretion in Melan-a cells [32]. Our studies for extracellular melanin in B16F10 cells were conducted with phenol-red-containing medium, which is routinely used for these cells; we conducted these assays based on published studies, which also used phenol-red-containing medium for extracellular assay measurements [33,34,35,36]. As our control group also consisted of phenol red, we believe that the interference of phenol red with absorbance values will not affect the results, which were reported as relative levels of melanin secreted as % of the control group. In addition, we confirmed that the interference was minimal by quantitating absolute levels of extracellular melanin using a calibration curve of synthetic melanin in phenol-red-containing medium (data not shown).

Even though the use of mushroom tyrosinase in lieu of mammalian tyrosinase has been validated as a model enzyme for screening tyrosinase inhibitors due to availability, a previous report indicated that mushroom and human tyrosinase exhibit differences in several molecular aspects [37]. Despite these limitations, our results demonstrated that AP showed similar effects in the case of fungal and human tyrosinases; AP did not affect both the mushroom tyrosinase in the cell-free system as well as human tyrosinase in HEMn-DP cells. Nevertheless, we noted that AP enhanced the activity against tyrosinase from B16F10 mouse melanoma cells where it showed inhibition of extracellular melanin. A previous study conducted with compound mulberroside A has also documented a similar finding where a large variation in the compound’s inhibitory capacity between mushroom and cellular tyrosinase from B16F10 cells was obtained [38]. Our results of cellular tyrosinase activity in B16F10 cells showed that AP significantly increased tyrosinase activity at concentrations (20–30 µM) where no change in melanin levels was noted, while, at the higher concentration of 50 µM where melanin was reduced, the activity was still upregulated, though it was not significantly different from the control. While at present, the mechanism of this is unknown, we speculate that this might be specific to the B16F10 tumor cell line, which has shown a biphasic response to compounds. The flavonoid quercetin, a nonsteroidal phytoestrogen, has also shown similar results in B16F10 cells where it increased cellular tyrosinase activity (with no change in cellular melanin content) at lower concentrations (5–20 µM), while at a higher concentration of 50 µM, the tyrosinase activity was decreased with reduction in melanin content [39]. Other flavonoids luteolin and taxifolin also increased cellular tyrosinase protein levels but reduced melanin [40]. We speculate that, similar to quercetin, the stimulation of cellular tyrosinase activity by AP at lower concentrations might be mediated by its prooxidant effects, which are involved in the redox cycling activity; however, this hypothesis warrants further testing.

As human melanocytes do not secrete detectable levels of melanin in the culture medium, any changes in extracellular melanin in HEMn-DP cells by compounds are confounded by cytotoxicity and, thus, lead to overestimation of the reduction in extracellular melanin. Due to this, dendricity was quantified as the marker for melanosome export in darkly pigmented (DP) cells; the dendrite lengths, number, and % distribution is quantified to provide an accurate estimate of the effects of the compound on melanosome export. Several reports have also documented the reduction in dendricity via the reduction in number and/or length as one of the targets for anti-melanogenic activity [41,42,43,44]. Our results of suppression of dendricity in the absence of the effects on both melanin synthesis and tyrosinase activity in cells are in agreement with several previous studies, which reported similar findings with compounds such as ginsenoside F1 (GF1) [44], centaureidin [42], and methylophiopogonanone B [43]. Also, the anti-melanogenic mechanism of AP is similar to niacinamide [45], as well as its analog N-nicotinoyl dopamine, due to its capacity to suppress melanosome export in the absence of effects on melanin synthesis and tyrosinase activity [46,47]. Interestingly, we noted that, in the study conducted on the niacinamide analog [46], the authors stated that the expression of melanogenesis proteins: Tyrosinase, MITF, TYR-1, and TYR-2, were unaffected, while other proteins related to melanosome export were not evaluated. Due to a few limitations, the current study only focused on melanogenic inhibition in the B16F10 and HEMn-DP cell model to provide proof-of-principle information of the depigmenting properties of AP; hence, the expression of melanogenic-related proteins to further elucidate anti-melanogenic mechanisms was not tested. The study of mechanisms of suppression of melanosome export by AP was not the focus of the present preliminary report; however, it would be very interesting in future studies to elucidate whether the mechanism of this is related, at least in part, to a downregulation in melanosome transport proteins.

We also confirmed that AP did not cause cytotoxicity to HaCaT cells at the concentrations at which it inhibited melanosome export in melanocytes. However, one of the limitations in our study is that only a cytotoxicity assay was conducted on HaCaT cells; hence, further testing of skin irritation and skin sensitization is warranted to establish the safety of AP as a skin-whitening agent for further development in clinical and cosmetic use.

## 4. Materials and Methods

### 4.1. Materials

Asoprisnil (J867, chemical purity 98%) was purchased from Axon Medchem (Reston, VA, USA). Kojic acid (KA), sodium phosphate monobasic powder, mushroom tyrosinase enzyme (T3824, lyophilized powder, ≥1000 U/mg solid), and L-dihydroxyphenylalanine (L-DOPA) were purchased from Sigma-Aldrich (St. Louis, MO, US). Dulbecco’s modified Eagle medium (Gibco™ DMEM, High glucose, pyruvate), TrypLE Express (1X), Dulbecco’s phosphate buffer saline (DPBS), and Penicillin-Streptomycin (10,000 U/mL) were purchased from Thermo Fisher Scientific (Waltham, MA, USA). Heat-inactivated fetal bovine serum (HI-FBS) was procured from R&D Systems Inc. (Minneapolis, MN, USA). Cell-lysis buffer (2X Cell Lysis Buffer, Cat #: EA-0001) was purchased from Signosis Inc. (Santa Clara, CA, USA).

### 4.2. Cell Culture

B16F10 mouse melanoma cells (CRL-6475™) were obtained from American Type Culture Collection (ATCC; Manassas, VA) and cultured using DMEM supplemented with 10% HI-FBS and 1% penicillin-streptomycin. Human keratinocytes (HaCaT) cells were obtained from AddexBio (San Diego, CA, US) and were cultured in DMEM with 10% HI-FBS and 1% penicillin-streptomycin. For all assays, HaCaT cells were used between Passage 18 and 20. Human melanocytes from darkly pigmented neonatal donor (HEMn-DP) were obtained from Cascade Biologics (Oregon, US) and cultured in Medium 254 (Cascade Biologics) supplemented with 1% Human Melanocyte Growth Supplement (HMGS) and 1% penicillin-streptomycin. HEMn-DP cells were used at passages between 5 and 10 for all assays. No antimycotic (amphotericin B) was used for supplementation for all cell cultures, and all cell cultures were routinely passaged for experiments using TrypLE Express reagent. All cells were maintained in a humidified atmosphere in a 95% air – 5% CO_2_ incubator at 37 °C.

### 4.3. Cytotoxicity Assay in B16F10 Cells

In order to test AP for effects on melanin synthesis, we first tested AP using MTS cytotoxicity assay (CellTiter Aqueous one, Promega Corp.) to identify nontoxic concentration ranges. B16F10 cells were seeded (5 × 10^3^ cells/well in 0.2 mL medium) in a 96-well tissue-culture plate (Corning) for 24 h at 37 °C in a 5% CO_2_ incubator. The final DMSO concentration in all groups including the control was 0.4%, which did not cause cytotoxicity. AP was added at various concentrations and cultures were maintained for a further duration of 72 h. At the end of the 72 h, the culture medium was aspirated and replaced by 100 μL of fresh media. MTS (20 μL) was added and incubated for 40 min, and the absorbance was read at 490 nm using a Versamax^®^ microplate reader. Cell viability was calculated from the absorbance values relative to control groups and expressed in %.

### 4.4. Extracellular and Intracellular Melanin Assay in B16F10 Cells

B16F10 cells were seeded at 6 × 10^4^ cells in 1.5 mL medium/well in 12-well plates and incubated for 24 h. After 24 h, the culture medium was replaced with fresh medium containing AP at various concentrations and cultured for another 72 h. At the end of the exposure duration, the culture medium was collected, centrifuged, and aliquoted in a 96-well plate, and its absorbance was measured at 475 nm using a microplate reader to estimate extracellular melanin. For the estimation of intracellular melanin, the cells remaining in wells were collected after detachment (1X TrypLE Express), and cell pellets were washed in DPBS. After aspiration, 250 μL of 1 N NaOH was added and heated to 70 °C to solubilize melanin. Next, 200 µL aliquots of lysate were transferred to a 96-well plate and the absorbance was read at 475 nm. A portion of the lysate was used to evaluate total protein content using bicinchoninic acid (BCA) assay (Pierce BCA kit, Thermo Fisher Scientific). Our method was based on measuring the absorbance of melanin (extracellular and intracellular) and normalizing it to total protein content; the ratio (Abs/µg protein) was expressed as % of the control group, which is similar to the method adopted in previous studies for measurement of melanin [48,49,50].

### 4.5. Intracellular Tyrosinase Activity in B16F10 Cells

We quantitated the cellular tyrosinase activity to delineate if the mechanisms of melanogenesis inhibition by AP might be related, at least in part, to a suppression of intracellular tyrosinase activity. B16F10 cells were cultured in 12-well tissue culture plates at a seeding density of 6 × 10^4^ cells/well. After 24 h, the culture medium was changed and AP was added, and further incubated for 72 h. At the end of the treatments, the cells were detached, and cell pellets were washed in DPBS and lysed in a 1X cell-lysis buffer, which contained 1% NP-40. Lysates (50 µL) were then aliquoted in a 96-well microplate, and 100 µL of a 3 mM solution of freshly prepared L-DOPA substrate solution was added. The absorbance was then measured using the kinetic mode at 475 nm every 30 s for 30 min at 30 °C using a microplate reader. The % tyrosinase activity was calculated from the slope of the linear range of the velocities of inhibition and was normalized to the total protein contents assayed by the BCA kit.

### 4.6. Mushroom Tyrosinase Activity

The direct effects of AP on tyrosinase activity were tested using a tyrosinase enzyme (isolated from mushrooms) with a L-DOPA substrate similar to the method reported in our earlier study [51]. Briefly, 80 µL of AP was prepared at different concentrations in 0.05 M sodium phosphate buffer (pH 6.5) and then added to a 96-well plate followed by 100 µL of freshly prepared substrate solution (6 mM L-DOPA in phosphate buffer). The reaction was started by adding 20 µL of mushroom tyrosinase enzyme (final concentration of enzyme in wells was 3.5 µg/mL). The production of dopachrome was monitored by measuring the kinetics of absorbance at 475 nm (for 10 min every 30 s). The slopes of the kinetic readings were calculated to determine and compare the tyrosinase activity from control.

### 4.7. Copper Chelation Assay

Copper-ion chelation activity was assayed by a pyrocatechol violet (PV) indicator assay based on the method reported in our earlier work [52]. Briefly, 100 µL of AP at different concentrations (prepared in a 50 mM sodium acetate buffer, pH 6.0) was aliquoted in a 96-well plate; kojic acid (KA; 500 µM) was used as a positive control and the negative control group consisted of buffer only. Next, 10 µL of 2 mM copper sulfate was added to the wells and incubated for 10 min followed by addition of 10 µL of 2 mM PV solution, and incubation continued for another 20 min. The absorbance was measured at 632 nm using a microplate reader and reported as % normalized to the control group.

### 4.8. Cytotoxicity Assay in Normal Melanocytes-Neonatal Darkly Pigmented (HEMn-DP) Cells

AP was further tested to evaluate its safety profile in human melanocytes obtained from neonatal darkly pigmented (DP) skin. Briefly, HEMn-DP cells were seeded at 1.4 × 10^4^ cells in 0.2 mL medium/well in 48-well plates for 72 h. At the end of the 72 h, the medium was aspirated and replaced by AP prepared at various doses in complete medium and cultured for a further duration of 5 d, with the compound replenished on the third day of treatment. After the treatments, MTS assay was conducted similar to the method outlined earlier by incubating a mixture of 20 μL of MTS dye with 100 μL of fresh media and incubated for 2 h, and the absorbance was read at 490 nm using the microplate reader. Cell viability was calculated from the absorbance values relative to the control.

### 4.9. Intracellular Melanin Assay in HEMn-DP Cells

HEMn-DP cells (8.5 × 10^4^ cells/well) were cultured in 12-well plates for 72 h followed by the replacement of medium with various nontoxic doses of AP and cultured for a further duration of 5 d. At the end of the treatments, the cell pellets were harvested and solubilized in 1 N NaOH and heated; the melanin levels were measured based on the method reported earlier in the case of B16F10 cells, and normalized to the total protein content and expressed as % of the control group.

### 4.10. Intracellular Tyrosinase Activity in HEMn-DP Cells

HEMn-DP cells (9 × 10^4^ cells/well) were cultured in 12-well plates for 72 h followed by addition of AP at various concentrations in 1 mL medium/well and cultured for a period of 5 d. After the incubation period, the cells were harvested and lysed; 25 µL of lysates was aliquoted in a 96-well plate with 50 µL of a 3 mM L-DOPA substrate, and the tyrosinase activity was monitored similar to the method reported earlier.

### 4.11. Melanosome Export Analysis by Dendricity

HEMn-DP cells (4.5 × 10^4^ cells/well) were cultured in six-well plates for 48 h followed by treatment with AP for 5 d. At the end of the treatments, the cells were imaged using a Nikon Labphot microscope at a 20X objective under phase-contrast mode, and random fields were imaged from each well using the NIS Elements 5.0 imaging software package. We estimated the total number of dendrites in each cell by manual counting and the total dendrite length (TDL) was estimated by measuring the dendrite lengths of each cell using the imaging software (NIS Elements 5.0) by digital tracing, after which these lengths were added to give the TDL. Third, the number of cells that had >3 dendrites were counted and expressed as the % of the total number of cells, which is similar to the parameter estimated previously [41,53].

### 4.12. Cytotoxicity Assay in Human Keratinocytes

AP was further tested to evaluate its safety profile in human keratinocytes, which form the epidermal-melanin unit with melanocytes in skin. Briefly, HaCaT cells were seeded at a density of 3.5 × 10^3^ cells in 0.2 mL medium/well in a 96-well plate and cultured for 48 h. At the end of the 48 h, the culture medium was replaced with fresh medium consisting of AP at various concentrations, and the cultures were maintained for 5 d. At the end of the treatment, MTS assay was conducted based on the method reported earlier.

### 4.13. Statistical Analysis

One-way analysis of variance (ANOVA) with Dunnett’s post-hoc test was run using GraphPad Prism version 8.0.0 for Windows, GraphPad Software, San Diego, California, US. Differences were considered statistically significant at *p* < 0.05. All data are reported as mean ± SEM.

## 5. Conclusions

In conclusion, our results demonstrate the potential of AP as an anti-melanogenic agent, as reflected in its capacity to significantly inhibit melanogenesis by mechanisms that may involve one or more steps in melanosome export. Our results thus establish a proof-of-principle for repurposing AP at low micromolar doses as a potential candidate for treatment of hyperpigmentation disorders. Further studies to elucidate the molecular mechanisms of AP are warranted. Additionally, studies to identify if other SPRMs might also exhibit a similar anti-melanogenic capacity like AP would also be worthy of future investigation.
